# Commentary: Recombinant *Mycobacterium tuberculosis* fusion protein for diagnosis of *Mycobacterium tuberculosis* infection: a short-term economic evaluation

**DOI:** 10.3389/fpubh.2026.1925611

**Published:** 2026-09-17

**Authors:** Chaoyuan Ma, Jin Li

**Affiliations:** 1 Hualan Biological Engineering Inc., Xinxiang, China; 2 Hualan Biological Vaccine Inc., Xinxiang, China

**Keywords:** asymmetric citation, cost per person, *Mycobacterium tuberculosis* fusion protein, pharmacoeconomic evaluation, tuberculin purified protein derivative, vial utilization

## 1 Introduction

Liu et al. (1) conducted the first head-to-head short-term economic evaluation comparing recombinant *Mycobacterium tuberculosis* fusion protein (EC) with tuberculin purified protein derivative (TB-PPD) for diagnosing *M. tuberculosis* infection from a Chinese societal perspective. The decision tree model framework and sensitivity analyses represent a commendable methodological effort. However, two concerns—the handling of per-vial and per-person test costs in the base-case analysis and the asymmetric citation of only one product's instruction—may affect the interpretation of the conclusions.

This commentary aims to: (a) question which utilization assumption should be selected for the base case, (b) request greater prominence for the scenario results, (c) recommend empirically informed vial-utilization and wastage parameters, and (d) request balanced citation of product information.

## 2 Per-vial cost vs. per-person cost: a methodological distinction

The base-case analysis used per-vial prices as model inputs for test cost inputs: EC at 98.00 CNY and TB-PPD at 136.78 CNY, concluding that EC was the dominant strategy with both lower cost and better utility. However, per the product instructions, each EC vial (0.3 ml) serves up to three subjects (2) and each TB-PPD vial (1 ml:50 IU) serves up to ten subjects (3). Under a maximum vial utilization (full-utilization) assumption, the per-person costs would be 32.67 CNY (98.00/3) for EC and 13.68 CNY (136.78/10) for TB-PPD—EC's per-person cost is approximately 2.4 times higher than TB-PPD's (Table 1).

**Table 1:** Impact of vial-utilization assumption on the economic evaluation conclusions.

Parameter	Per-vial (base-case)	Full-utilization (scenario)
EC test cost	98.00 CNY	32.67 CNY
TB-PPD test cost	136.78 CNY	13.68 CNY
EC cost relative to TB-PPD	Lower (0.72 × )	Higher (2.39 × )
Incremental cost (EC—TB-PPD)	−960.22 CNY	−902.45 CNY
Incremental QALYs (EC—TB-PPD)	+0.0050	+0.0050
Cost-utility (ICUR)	EC dominant (ICUR = −192,043.60 CNY/QALY)	EC dominant (ICUR = −180,489.60 CNY/QALY)
Cost-effectiveness on misdiagnosis rate (ICER)	EC dominant (ICER = −7,263.53 CNY per percentage-point reduction)	Reversed (ICER = +3,556.85 CNY per percentage-point reduction)
Acquisition cost per test	EC lower (98.00 vs. 136.78 CNY)	TB-PPD lower (13.68 vs. 32.67 CNY)
Total modeled cost	EC lower (579.05 vs. 1,539.27 CNY)	EC lower (513.72 vs. 1,416.17 CNY)
Conclusion	EC remains dominant in the cost-utility model under both costing approaches (lower total modeled cost and higher QALYs);	Under the full-utilization assumption, TB-PPD has the lower acquisition cost per test.

EC, Recombinant *Mycobacterium tuberculosis* fusion protein; TB-PPD, tuberculin purified protein derivative; ICUR, incremental cost-utility ratio; ICER, incremental cost-effectiveness ratio; QALY, quality-adjusted life year. The “full-utilization (scenario)” column assumes maximum vial sharing (EC: 3 persons/vial; TB-PPD: 10 persons/vial) and therefore represents a lower-bound per-test acquisition cost under an idealized assumption, not the definitively correct per-person cost in routine practice. Incremental values are calculated as EC minus TB-PPD; a negative incremental cost indicates that EC has the lower total modeled cost, and a positive incremental QALY value indicates that EC yields more QALYs. A negative ICUR (southeast quadrant) denotes dominance—that is, EC has both lower total cost and higher QALYs; because its magnitude is sensitive to the small QALY difference, the incremental cost and incremental QALYs are reported separately above. “Acquisition cost per test” refers to reagent purchase cost only; “total modeled cost” includes downstream confirmatory testing and treatment.

It is important to distinguish among: (a) acquisition price per vial, (b) provider charge per patient encounter, (c) expected resource cost per completed test, (d) unused-dose wastage, and (e) other administration and consumable costs. The original base-case used the per-vial acquisition price as a proxy for the per-patient charge or the expected per-test cost. The original scenario analysis, described in Methods Section 2.5, Results Section 3.3, and Table 2 of the main article, represented a maximum-utilization assumption.

The original authors' pragmatic justification—that hospitals may charge per vial due to uncertain patient volumes—is understandable in practice but inadequate in modeling. A rigorous approach would estimate expected subjects per vial under real-world conditions and derive actual per-person costs accordingly, presenting both scenarios in the base-case with equal prominence.

## 3 Asymmetric citation of product information

The original study cited the EC product instruction (2) to support its arguments (1) but omitted the TB-PPD product instruction (3). While this asymmetry does not alter the model's sensitivity, specificity, cost, or utility parameters, citing both instructions would improve transparency. The TB-PPD instruction specifies three indications: (i) clinical diagnosis of tuberculosis, (ii) selection of BCG vaccination subjects, and (iii) monitoring of immune response after BCG vaccination (3). EC's instruction specifies only one: auxiliary diagnosis of *M. tuberculosis* infection (2). This reflects different design rationales: TB-PPD contains BCG-shared antigens enabling BCG-related functions but yielding low specificity (~26.58%) in vaccinated populations; EC uses ESAT-6/CFP-10 antigens absent in BCG, achieving high specificity (~92.72%) in the phase III trial reported in the original study (1, 2) but cannot serve BCG-related purposes (4). These performance differences are consistent with the broader evidence base on *M. tuberculosis* antigen-based skin test (5). We note that the original decision problem was specifically the diagnosis of *M. tuberculosis* infection, so excluding BCG-related functions was not inherently biased. However, transparent reporting should cite both product instructions and state the intended clinical scope. The omission of the TB-PPD instruction does not necessarily imply that model parameters were affected, but it does limit the reader's ability to assess the completeness of the comparison.

## 4 Discussion

Liu et al. (1) made a valuable contribution as the first economic comparison of EC and TB-PPD in China. The finding that EC's higher specificity reduces misdiagnosis-related downstream costs is clinically meaningful. A subsequent long-term evaluation by the same group confirmed EC's cost-utility advantage over a 77-year horizon (6). Recent evidence further demonstrates that economic conclusions depend substantially on the target population, time horizon, and screening pathway: in older diabetic patients, traditional TST had a high probability of being the most cost-effective screening strategy (7), and WHO's assessment of *M. tuberculosis* antigen-based skin tests noted that cost-effectiveness varies by setting (8). However, the base-case dominance conclusion should be interpreted with the following caveats: When per-person costs under maximum-utilization assumptions are applied, EC remains dominant in the cost-utility analysis (lower total modeled cost and higher QALYs) but no longer dominates on cost-effectiveness for the misdiagnosis rate. The scenario results, while reported in the main article, should be given equal prominence in the conclusions. Future economic evaluations of multi-dose vial diagnostic products should report resource-use assumptions, costing methods, and uncertainty transparently in line with the CHEERS 2022 statement (9). Specifically, they should: (i) calculate per-person costs as the primary model input; (ii) estimate real-world vial utilization and wastage rates; (iii) present both per-vial and per-person scenarios in the base-case; and (iv) cite all relevant product instructions and clearly define the comparison scope. When comparing products with different approved indications, both product instructions should be cited, and the comparison scope should be transparently defined to ensure a balanced and impartial synthesis of evidence.

## References

[1] Liu Z Diao S Zeng L Liu D Jiao X Chen Z . Recombinant *Mycobacterium tuberculosis* fusion protein for diagnosis of *Mycobacterium tuberculosis* infection: a short-term economic evaluation. Front Public Health. (2023) 11:1105857. doi: 10.3389/fpubh.2023.1105857 37206861 PMC10190601

[2] Zhifei Longcom Biologic Pharmacy Co. Instruction of Recombinant Mycobacterium Tuberculosis Fusion Protein (EC) (2020). Available online at: https://zy.yaozh.com/instruct/sms20211020/58.pdf (Accessed September 6, 2026).

[3] Beijing Simcere Sanroad Biologicals Co. Ltd. Instruction of Tuberculin Purified Protein Derivative (TB-PPD). Available online at: http://www.sanroadbio.com/attached/upload/file/20250902/6389243026651380969200272.jpg (Accessed August 22, 2026).

[4] Wu J Lu AD Zhang LP Zuo YX Jia YP. Study of clinical outcome and prognosis in pediatric core binding factor-acute myeloid leukemia. Zhonghua Xue Ye Xue Za Zhi. (2019) 40:52–7. Chinese. doi: 10.3760/cma.j.issn.0253-2727.2019.01.010 30704229 PMC7351698

[5] Krutikov M Faust L Nikolayevskyy V Hamada Y Gupta RK Cirillo D . The diagnostic performance of novel skin-based *in-vivo* tests for tuberculosis infection compared with purified protein derivative tuberculin skin tests and blood-based *in vitro* interferon-γ release assays: a systematic review and meta-analysis. Lancet Infect Dis. (2022) 22:250–64. doi: 10.1016/S1473-3099(21)00261-9 34606768

[6] Diao S Liu Z Liu D Cheng X Zeng L Jiao XF . Long-term economic evaluation of the recombinant *Mycobacterium tuberculosis* fusion protein (EC) test for the diagnosis of *Mycobacterium tuberculosis* infection. Front Pharmacol. (2023) 14:1161526. doi: 10.3389/fphar.2023.1161526 37261290 PMC10228647

[7] Song HW Lin Y Wan TE Zhang CF Pan JS. Cost-effectiveness analysis of tuberculosis screening in diabetic patients in China: a decision-analytic Markov model. Front Public Health. (2026) 13:1696952. doi: 10.3389/fpubh.2025.1696952 41561859 PMC12813112

[8] World Health Organization. Rapid Communication: TB Antigen-Based Skin Tests for the Diagnosis of TB Infection. Geneva: World Health Organization (2022). Available online at: https://www.who.int/publications/i/item/WHO-UCN-TB-2022.1 (Accessed September 6, 2026).

[9] Husereau D Drummond M Augustovski F de Bekker-Grob E Briggs AH Carswell C . Consolidated health economic evaluation reporting standards 2022 (CHEERS 2022) statement: updated reporting guidance for health economic evaluations. Value Health. (2022) 25:3–9. doi: 10.1016/j.jval.2021.11.1351 35031096

